# Prediction of the gait function using the nigrostriatal and corticoreticulospinal tracts of the affected hemisphere in a cerebral infarct: A diffusion tensor imaging study

**DOI:** 10.1097/MD.0000000000030788

**Published:** 2022-09-30

**Authors:** Sung Ho Jang, Sang Seok Yeo, Min Jye Cho

**Affiliations:** a Department of Physical Medicine and Rehabilitation, College of Medicine, Yeungnam University, Namku, Taegu, Republic of Korea; b Department of Physical Therapy, College of Health Sciences, Dankook University, Dongnamgu, Cheonan, Republic of Korea.

**Keywords:** corticoreticulospinal tract, diffusion tensor imaging, diffusion tensor tractography, Gait function, nigrostriatal tract

## Abstract

Prognosis predictability of the nigrostriatal tract (NST) and corticoreticulospinal tract (CRT) of affected hemisphere at early stage for gait function at chronic stage were investigated using diffusion tensor tractography (DTT) in patients with a cerebral infarction.

Thirty consecutive patients with middle cerebral artery (MCA) territory infarction were recruited. Functional ambulation category (FAC) was used to evaluate the gait function at chronic stage. Fractional anisotropy (FA) and tract volume (TV) of ipsilesional NST and ipsilesional CRT were determined to be DTT parameters at early stage.

FAC score at chronic stage showed strong positive correlations with TVs of ipsilesional NST and ipsilesional CRT at early stage (ipsilesional NST *R* = 0.786; ipsilesional CRT *R* = 0.821; *P* < .05). According to regression model, FAC score at chronic stage was positively related to TVs of ipsilesional NST and ipsilesional CRT at early stage (Adjusted *R*^2^ = 0.700, *F* = 34.905, *P* < .05). FAC score at chronic stage was associated more positively with TV of ipsilesional CRT (β = 0.532) than that of ipsilesional NST (β = 0.362).

Ipsilesional NST and ipsilesional CRT at early stage had prognosis predictability for gait function at chronic stage in patients with an MCA infarction. Moreover, ipsilesional CRT had stronger predictability than ipsilesional NST.

## 1. Introduction

Stroke is one of the major causes of adult disability, with gait dysfunction being one of the most serious disabling sequelae of stroke. Approximately 20 to 30% of stroke patients do not recover their ability to walk.^[1,2]^ Gait dysfunction adversely affects mobility, activities of daily living, and health.^[3]^ Therefore, a precise prediction of the prognosis of gait function at the early stages of stroke is clinically important because it could provide helpful information for establishing specific rehabilitation strategies and estimating the duration of rehabilitation.

Among the neural tracts for the motor function, the corticoreticulospinal tract (CRT) plays an important role in gait by controlling the axial and proximal muscles of the limbs.^[4,5]^ Many studies reported the relationship between the gait function and CRT in stroke patients.^[6-11]^ Previous studies suggested that the nigrostriatal tract (NST) is also involved in gait function by affecting voluntary movement through the basal ganglia, which control movement during gait.^[12,13]^ The NST is one of the major dopaminergic pathways that originate from the substantia nigra pars compacta in the midbrain and terminate at the dorsal striatum. A few studies reported the relationship between the severity of gait impairment and reduced dopamine availability in the nigrostriatal system in patients with Parkinson’s disease and multiple system atrophy.^[14,15]^

Developments in diffusion tensor tractography (DTT), which is derived from diffusion tensor imaging (DTI), enable a three-dimensional reconstruction and visualization of the NST and CRT.^[16,17]^ A few studies attempted to demonstrate the prognosis predictability of the CRT on gait function of stroke patients using DTT.^[8,11]^ By contrast, no study of the NST on this topic has been reported.

In this study, DTT was used to investigate the prognosis predictability of the NST and CRT of the affected hemisphere at the early stage for the gait function at the chronic stage in patients with a cerebral infarction.

## 2. Methods

### 2.1. Subjects

Thirty consecutive patients with middle cerebral artery (MCA) territory infarction (15 men, 15 women; mean age 56.93 ± 10.55 years; range, 34–68 years) were recruited according to the following inclusion criteria: first-ever stroke; age: 20 to 69 years; hemiparesis contralateral to infarct lesion; MCA territory infarct confirmed by a neuroradiologist; an inability to walk independently (functional ambulation category [FAC] ≤ 3) at the early stage (within 1 month after onset); gait function evaluation was performed at the chronic stage (more than three months after onset); DTI scanning was performed at the early stage; no prior history of head trauma, psychiatric, and neurological disease. This study was performed retrospectively and conducted in accordance with the guideline of the Declaration of Helsinki. All of the patients provided signed, informed consent and the study protocol was approved by the institutional review board of the Yeungnam University Hospital (IRB number: YUMC 2021-03-014).

### 2.2. Gait function evaluation

The FAC scale was used to evaluate the walking ability at the chronic stage (mean: 7.65 ± 3.12 months) after stroke onset.^[18]^ This 6-point scale is assessed to determine the levels of support needed during a 15 m walk.^[9]^ The FAC was categorized as follows: 0 (nonfunctional ambulation); 1 (requires firm continuous support from one person); 2 (requires continuous or intermittent support from 1 person); 3 (requires verbal supervision or stand-by help); 4 (can walk independently except help on stairs and uneven surfaces); 5 (can walk independently anywhere).^[9]^ The reliability and validity of the FAC are well-established.^[18]^

### 2.3. Diffusion tensor imaging and tractography

**Figure 1.**
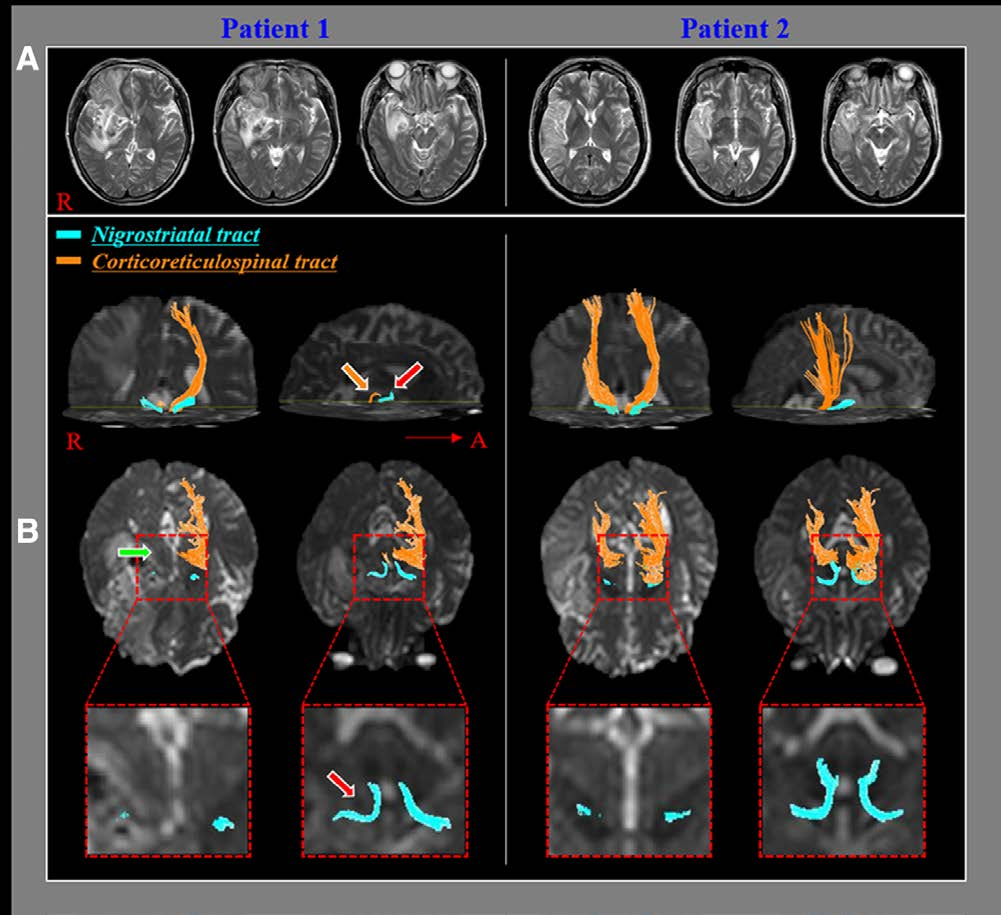
Results of diffusion tensor tractography for the nigrostriatal tract (NST) and the corticoreticulospinal tract (CRT). (A) T2-weighted brain magnetic resonance images at the time of diffusion tensor imaging scanning in representative patients with a middle cerebral artery territory infarction (patient 1:51-year-old female with functional ambulation category [FAC] score 2 at the chronic stage, patient 2: 67-year-old female with FAC score 5 at the chronic stage). (B) The ipsilesional NST (red arrow) and the ipsilesional CRT (orange arrow) at the early stage in patient 1 have a lower tract volume than those in patient 2. The ipsilesional CRT (green arrow) at the early stage in patient 1 shows discontinuation at the lesion. By contrast, preservation of the integrity of the ipsilesional CRT at the early stage is observed in patient 2.

The DTI data were obtained at the early stage (mean: 12.43 ± 5.65 days) after stroke onset. DTI was performed using a sensitivity-encoding head coil on a 1.5 T Philips Gyroscan Intera (Hoffman-LaRoche Ltd., Best, Netherlands) scanner with single-shot echo-planar imaging and navigator echo. Sixty-seven contiguous slices (acquisition matrix = 96 × 96; reconstruction matrix = 192 × 192; field of view = 240 mm × 240 mm; TR = 10,726 ms; TE = 76 ms, b = 1000 s/mm^2^, NEX = 1, and thickness = 2.5 mm) were acquired for each of the 32 noncollinear diffusion-sensitizing gradients. Eddy current image distortion and head motion effects were adjusted using affine multi-scale 2-dimensional registration from the Oxford Centre for Functional Magnetic Resonance Imaging of Brain (FMRIB) Software Library (FSL: www.fmrib.ox.ac.uk/fsl).^[19]^ The NST and CRT were assessed using a DTI Studio software (CMRM, Johns Hopkins Medical Institute, Baltimore, Maryland) based on the fiber assignment continuous tracking (FACT) algorithm. The NST was tracked by manually assigning the first regions of interest (ROI) on the substantia nigra at the midbrain on the fractional anisotropy (FA) map. The second ROI was located on the striatum on the FA map.^[20,21]^ The CRT was reconstructed by placing the first and second ROI manually on the reticular formation of the medulla and the tegmentum of the midbrain, respectively.^[16]^ Fiber tracking of the NST was started at the center of a seed voxel with FA > 0.1 and ended at a voxel with FA > 0.1 and a tract turning-angle <70° with an option of cut operation on the axial images (Fig. 1).^[17,19]^ On the other hand, fiber tracking of the CRT was started at the center of the voxel with FA > 0.2 and ended at the voxel with FA of < 0.2 and a tract turning-angle < 60°.^[7]^

### 2.4. Statistical analysis

Statistical analysis was performed using SPSS 21.0 for Windows (SPSS, Chicago, IL). The Kolmogorov–Smirnov test and Shapiro–Wilk test were conducted to determine the normality of the FAC score at the chronic stage and DTT (FA, tract volume [TV]) parameters of the ipsilesional NST and the ipsilesional CRT at the early stage. The DTT parameters of the ipsilesional CRT did not satisfy normality (*P *< .05). According to the normality of the data, the Pearson correlation analysis was used to evaluate the significance of the correlations of the FAC score at the chronic stage with the DTT parameters of the ipsilesional NST and the ipsilesional CRT at the early stage of the patients. The correlation coefficient represents the strength of the relationship between two variables (0.10–0.29: weak correlation; 0.30–0.49: moderate correlation; more than 0.50: strong correlation).^[22]^ Based on correlation analysis, multiple linear regression analysis was performed to evaluate the DTT parameters at the early stage as the independent variables that predicted the FAC score at the chronic stage as the dependent variable.

## 3. Results

Table 1 lists the results of correlation analysis between the FAC score at the chronic stage and DTT parameters of the ipsilesional NST and the ipsilesional CRT at the early stage. The FAC score at the chronic stage had no significant correlation with the FA values of the ipsilesional NST and the ipsilesional CRT at the early stage (*P* > .05). By contrast, the FAC score at the chronic stage showed strong positive correlations with the TVs of the ipsilesional NST and the ipsilesional CRT at the early stage (ipsilesional NST *R *= 0.786; ipsilesional CRT *R *= 0.821; *P* < .05). Table 2 lists the results of multiple linear regression analysis between the FAC score at the chronic stage and DTT parameters of the ipsilesional NST and the ipsilesional CRT at the early stage. The FAC score at the chronic stage was positively related with the TVs of the ipsilesional NST and the ipsilesional CRT at the early stage (Adjusted *R*^2^ = 0.700, *F* = 34.905, *P* < .05). Moreover, the FAC score at the chronic stage was associated more positively with the TV of the ipsilesional CRT (β = 0.532) than that of the ipsilesional NST (β = 0.362).

**Table 1:** Correlations of the gait function at the chronic stage with diffusion tensor tractography parameters of the ipsilesional nigrostriatal tract and the ipsilesional corticoreticulospinal tract at an early stage.

	NST		CRT	
	FA	TV	FA	TV
FAC	0.228	0.786	0.295	0.821
	0.23	0.00*	0.11	0.00*

CRT = corticoreticulospinal tract, FA = fractional anisotropy, FAC = functional ambulation category, NST = nigrostriatal tract, TV = tract volume.

* Correlation is significant at *P* < .05.

**Table 2:** Multiple linear regression analysis between the gait function at the chronic stage and diffusion tensor tractography parameters of the ipsilesional nigrostriatal tract and the ipsilesional corticoreticulospinal tract at an early stage.

Dependent variable	Independent variable	B	se	β	*t*	*P*	VIF
FAC	NST TV	0.013	0.006	0.362	2.150	0.04*	2.744
	CRT TV	0.001	0.000	0.532	3.160	0.00*	2.744
	Adjusted R^2^ = 0.700 F = 34.905 *P* = .00*						

CRT = corticoreticulospinal tract, FAC = functional ambulation category, NST = nigrostriatal tract, VIF = variation inflation factor, TV = tract volume.

* Statistically significant at *P* < .05.

## 4. Discussion

DTT was used to examine the predictability of the ipsilesional NST and the ipsilesional CRT at the early stage for the gait function at the chronic stage in patients with an MCA infarction. The results can be summarized as follows. First, the FAC score at the chronic stage had strong positive correlations with the TVs of the ipsilesional NST and the ipsilesional CRT at the early stage. Second, the regression model showed that the FAC score at the chronic stage was positively associated with the TVs of the ipsilesional NST and ipsilesional CRT at the early stage. In particular, the FAC score at the chronic stage had a more positive relationship with the TV of the ipsilesional CRT than that of the ipsilesional NST.

The FA value represents the degree of directionality of water molecule diffusion and the integrity of white matter microstructures, including axons, myelin, and microtubules. Therefore, it reflects the fiber density, axonal diameter, and white matter myelination.^[23,24]^ The TV indicates the volume of voxels within the neural tract representing the number of neural fibers.^[24,25]^ The FAC score at the chronic stage showed strong positive correlations with the TVs of the ipsilesional NST and the ipsilesional CRT at the early stage. This indicates that the gait function at the chronic stage was closely related to the remaining fiber number of ipsilesional NST and ipsilesional CRT at the early stage. These results suggest that the remaining fiber number of the ipsilesional NST and the ipsilesional CRT at the early stage could predict the gait function at the chronic stage in patients with an MCA infarction.

Regarding the regression model, the TVs of the ipsilesional NST and the ipsilesional CRT at the early stage affected the FAC score at the chronic stage. In addition, the TV of the ipsilesional CRT at the early stage had a larger effect on the FAC score at the chronic stage than that of the ipsilesional NST at the early stage. Consequently, the remaining fiber number of the ipsilesional CRT at the early stage has more predictability on the gait function at the chronic stage than that of the ipsilesional NST at the early stage in patients with an MCA infarction.

The nigrostriatal dopaminergic projection was reported to facilitate the initiation of gait by increasing the excitability of the dopamine D1-expressing neurons through the direct pathway and reducing the tonic inhibition sent by the output from the basal ganglia to the mesencephalic locomotor region.^[26]^ On the other hand, it could also help disinhibit the activity of the mesencephalic locomotor region by decreasing the excitability of the dopamine D2 expressing neurons through the indirect pathways.^[27,28]^ As a result, input to the mesencephalic locomotor region controls the gait and posture through projects to the spinal motor neurons via the reticulospinal tract.^[29]^ A few studies reported a relationship between the gait function and the nigrostriatal system in patients with neurological diseases.^[14,15]^ Brooks et al reported that the severity and duration of the gait dysfunction were correlated with the loss of the nigrostriatal dopaminergic system in the putamen in patients with Parkinson disease and multiple system atrophy.^[14]^ Ouchi et al used positron emission tomography to confirm that the reduction of walking cadence was associated with a dopamine deficit in the nigrostriatal projection area, particularly the caudate in patients with Parkinson’s disease.^[15]^

A few studies reported the predictability of the CRT in stroke patients using DTT, but the results were controversial.^[8,11^ Soulard et al reported that the walking score at 1 month to 2 years after onset was correlated with the FA value of the ipsilesional CRT at the baseline (1 month after onset) in patients with a supratentorial infarction.^[8]^ By contrast, Jun et al reported that the FAC score at 6 months after onset was not related to the FA value and TV of the CRT at 1 month after onset in patients with a supratentorial stroke (hemorrhage and infarction).^[11]^ On the other hand, the above studies did not recruit only the patients who could not walk independently at the early stage of stroke, as in the present study. Consequently, to the best of the authors’ knowledge, this study is the first to demonstrate the predictability of both the ipsilesional NST and the ipsilesional CRT on the gait function in patients with cerebral infarction.

This study had some limitations. First, DTT analysis produced false-positive and false-negative results primarily due to crossing fibers or the partial volume effect.^[30]^ Second, because this study was performed retrospectively, only the FAC score was used to evaluate the gait function. Third, although the NST and CRT are related to the gait function, there are other neural tracts, such as the corticospinal tract or cortico-pontocerebellar tract associated with the gait function that could be affected by an MCA infarct. Fourth, the clinical features could be more severe than those in a general population of chronic ischemic stroke patients because the patients who visited the rehabilitation department of a university hospital were recruited in this study. Hence, further prospective studies on various gait function evaluations and additional neural tracts related to the gait function considering the generality of the clinical features are needed.

In conclusion, DTT showed that the ipsilesional NST and the ipsilesional CRT at the early stage had prognostic predictability for the gait function at the chronic stage in patients with an MCA infarction. In addition, the ipsilesional CRT had stronger predictability on gait function than the ipsilesional NST. These results suggest that the ipsilesional NST and the ipsilesional CRT states on DTT at the early stage can be used to predict the prognosis of the gait function in patients with an MCA infarction. Nevertheless, further studies of various brain pathologies, including an intracerebral hemorrhage, will be needed.

## Acknowlegdements

This research was supported by Basic Science Research Program through the National Research Foundation of Korea (NRF), funded by the Ministry of Education, Science and Technology (2021R1A2C1095047). Jang SH and Yeo SS contributed equally to this work and should be considered as co-first authors.

## Author contributions

**Conceptualization:** Sung Ho Jang, Sang Seok Yeo, Min Jye Cho.

**Data curation:** Sung Ho Jang, Min Jye Cho.

**Formal analysis:** Sang Seok Yeo, Min Jye Cho.

**Investigation:** Sung Ho Jang, Sang Seok Yeo, Min Jye Cho.

**Writing – original draft:** Min Jye Cho.

**Writing – review & editing:** Sung Ho Jang.

## References

[1] Wade DT Wood VA Hewer RL. Recovery after stroke – the first 3 months. J Neurol Neurosurg Psychiatry. 1985;48:7–13. 3973623 10.1136/jnnp.48.1.7 PMC1028175

[2] Freund HJ. Premotor area and preparation of movement. Rev Neurol (Paris). 1990;146:543–7. 2263816

[3] Fritz S Lusardi M. White paper: “walking speed: the sixth vital sign”. J Geriatr Phys Ther. 2009;32:46–9. 20039582

[4] Kably B Drew T. Corticoreticular pathways in the cat. I. Projection patterns and collaterization. J Neurophysiol. 1998;80:389–405. 9658059 10.1152/jn.1998.80.1.389

[5] Matsuyama K Mori F Nakajima K. Locomotor role of the corticoreticular-reticulospinal-spinal interneuronal system. Prog Brain Res. 2004;143:239–49. 14653169 10.1016/S0079-6123(03)43024-0

[6] Jang SH Chang CH Lee J. Functional role of the corticoreticular pathway in chronic stroke patients. Stroke. 2013;44:1099–104. 23444306 10.1161/STROKEAHA.111.000269

[7] Yoo JS Choi BY Chang CH. Characteristics of injury of the corticospinal tract and corticoreticular pathway in hemiparetic patients with putaminal hemorrhage. BMC Neurol. 2014;14:121. 24903632 10.1186/1471-2377-14-121 PMC4096439

[8] Soulard J Huber C Baillieul S. Motor tract integrity predicts walking recovery: a diffusion MRI study in subacute stroke. Neurology. 2020;94:e583–93. 31896618 10.1212/WNL.0000000000008755

[9] Yeo SS Jang SH Park GY. Effects of injuries to descending motor pathways on restoration of gait in patients with pontine hemorrhage. J Stroke Cerebrovasc Dis. 2020;29:104857. 32409256 10.1016/j.jstrokecerebrovasdis.2020.104857

[10] Jang SH Cho MK. Relationship of recovery of contra-lesional ankle weakness with the corticospinal and corticoreticular tracts in stroke patients. Am J Phys Med. 2022;101:659–65. 10.1097/PHM.0000000000001881 PMC9197145 35706118

[11] Jun S Hong B Kim Y. Does motor tract integrity at 1 month predict gait and balance outcomes at 6 months in stroke patients? Brain Sci. 2021;11:867. 34210075 10.3390/brainsci11070867 PMC8301763

[12] Mink JW Thach WT. Basal ganglia motor control. I. Nonexclusive relation of pallidal discharge to five movement modes. J Neurophysiol. 1991;65:273–300. 2016642 10.1152/jn.1991.65.2.273

[13] Ikemoto S. Brain reward circuitry beyond the mesolimbic dopamine system: a neurobiological theory. Neurosci Biobehav Rev. 2010;35:129–50. 20149820 10.1016/j.neubiorev.2010.02.001 PMC2894302

[14] Brooks DJ Salmon EP Mathias CJ. The relationship between locomotor disability, autonomic dysfunction, and the integrity of the striatal dopaminergic system in patients with multiple system atrophy, pure autonomic failure, and Parkinson’s disease, studied with PET. Brain. 1990;113(Pt 5):1539–52. 2123119 10.1093/brain/113.5.1539

[15] Ouchi Y Kanno T Okada H. Changes in dopamine availability in the nigrostriatal and mesocortical dopaminergic systems by gait in Parkinson’s disease. Brain. 2001;124:784–92. 11287377 10.1093/brain/124.4.784

[16] Yeo SS Chang MC Kwon YH. Corticoreticular pathway in the human brain: diffusion tensor tractography study. Neurosci Lett. 2012;508:9–12. 22197953 10.1016/j.neulet.2011.11.030

[17] Yeo SS Seo JP. The nigrostriatal tract between the substantia nigra and striatum in the human brain: a diffusion tensor tractography study. . J Kor Phys Ther. 2020;32:388–90.

[18] Mehrholz J Wagner K Rutte K. Predictive validity and responsiveness of the functional ambulation category in hemiparetic patients after stroke. Arch Phys Med Rehabil. 2007;88:1314–9. 17908575 10.1016/j.apmr.2007.06.764

[19] Smith SM Jenkinson M Woolrich MW. Advances in functional and structural MR image analysis and implementation as FSL. Neuroimage. 2004;23(Suppl 1):S208–219. 15501092 10.1016/j.neuroimage.2004.07.051

[20] Song YJ Korgaonkar MS Armstrong LV. Tractography of the brainstem in major depressive disorder using diffusion tensor imaging. PLoS One. 2014;9:e84825. 24465436 10.1371/journal.pone.0084825 PMC3897382

[21] Andica C Kamagata K Hatano T. Neurite orientation dispersion and density imaging of the nigrostriatal pathway in Parkinson’s disease: retrograde degeneration observed by tract-profile analysis. Parkinsonism Relat Disord. 2018;51:55–60. 29525556 10.1016/j.parkreldis.2018.02.046

[22] Cohen J. Statistical power analysis for the behavioral sciences. 2nd ed. Hillsdale, NJ: L. Erlbaum Associates, 1988.

[23] Mori S Crain BJ Chacko VP. Three-dimensional tracking of axonal projections in the brain by magnetic resonance imaging. Ann Neurol. 1999;45:265–9. 9989633 10.1002/1531-8249(199902)45:2<265::aid-ana21>3.0.co;2-3

[24] Assaf Y Pasternak O. Diffusion tensor imaging (DTI)-based white matter mapping in brain research: a review. J Mol Neurosci. 2008;34:51–61. 18157658 10.1007/s12031-007-0029-0

[25] de Groot M Ikram MA Akoudad S. Tract-specific white matter degeneration in aging: the Rotterdam study. Alzheimers Dement. 2015;11:321–30. 25217294 10.1016/j.jalz.2014.06.011

[26] Ryczko D Dubuc R. Dopamine and the brainstem locomotor networks: from lamprey to human. Front Neurosci. 2017;11:295. 28603482 10.3389/fnins.2017.00295 PMC5445171

[27] Albin RL Young AB Penney JB. The functional anatomy of basal ganglia disorders. Trends Neurosci. 1989;12:366–75. 2479133 10.1016/0166-2236(89)90074-x

[28] Kravitz AV Freeze BS Parker PR. Regulation of parkinsonian motor behaviours by optogenetic control of basal ganglia circuitry. Nature. 2010;466:622–6. 20613723 10.1038/nature09159 PMC3552484

[29] Takakusaki K. Functional neuroanatomy for posture and gait control. J Mov Disord. 2017;10:1–17. 28122432 10.14802/jmd.16062 PMC5288669

[30] Yamada K Sakai K Akazawa K. MR tractography: a review of its clinical applications. Magn Reson Med Sci. 2009;8:165–74. 20035125 10.2463/mrms.8.165

